# High Carbohydrate Diet Increased Glucose Transporter Protein Levels in Jejunum but Did Not Lead to Enhanced Post-Exercise Skeletal Muscle Glycogen Recovery

**DOI:** 10.3390/nu13072140

**Published:** 2021-06-22

**Authors:** Yumiko Takahashi, Yutaka Matsunaga, Hiroki Yoshida, Terunaga Shinya, Ryo Sakaguchi, Hideo Hatta

**Affiliations:** 1 Department of Sports Sciences, The University of Tokyo, 3-8-1 Komaba, Meguro, Tokyo 153-8902, Japan; y_matsunaga@idaten.c.u-tokyo.ac.jp (Y.M.); yoshida-oizumi1445@g.ecc.u-tokyo.ac.jp (H.Y.); terunagashinya@g.ecc.u-tokyo.ac.jp (T.S.); r.sakaguchi.uttf800@gmail.com (R.S.); hatta@idaten.c.u-tokyo.ac.jp (H.H.); 2 Department of Sport Research, Japan Institute of Sports Sciences, 3-15-1 Nishigaoka, Kita, Tokyo 115-0056, Japan

**Keywords:** glycogen recovery, high carbohydrate diet, jejunum, SGLT1, glucose absorption, PDH

## Abstract

We examined the effect of dietary carbohydrate intake on post-exercise glycogen recovery. Male Institute of Cancer Research (ICR) mice were fed moderate-carbohydrate chow (MCHO, 50%cal from carbohydrate) or high-carbohydrate chow (HCHO, 70%cal from carbohydrate) for 10 days. They then ran on a treadmill at 25 m/min for 60 min and administered an oral glucose solution (1.5 mg/g body weight). Compared to the MCHO group, the HCHO group showed significantly higher sodium-D-glucose co-transporter 1 protein levels in the brush border membrane fraction (*p* = 0.003) and the glucose transporter 2 level in the mucosa of jejunum (*p* = 0.004). At 30 min after the post-exercise glucose administration, the skeletal muscle and liver glycogen levels were not significantly different between the two diet groups. The blood glucose concentration from the portal vein (which is the entry site of nutrients from the gastrointestinal tract) was not significantly different between the groups at 15 min after the post-exercise glucose administration. There was no difference in the total or phosphorylated states of proteins related to glucose uptake and glycogen synthesis in skeletal muscle. Although the high-carbohydrate diet significantly increased glucose transporters in the jejunum, this adaptation stimulated neither glycogen recovery nor glucose absorption after the ingestion of post-exercise glucose.

## 1. Introduction

Glycogen is a primary energy source for skeletal muscle movement and exercise. Fatigue during prolonged moderate- to high-intensity exercise is associated with glycogen depletion [1]. Stimulating post-exercise glycogen repletion in skeletal muscle is one of the most important things to optimize an individual’s performance in subsequent exercise. Post-exercise skeletal muscle glycogen recovery is influenced by various factors. For example, carbohydrate ingestion could be critical for enhancing glycogen recovery. It has been consistently reported that carbohydrate intake clearly stimulates glycogen repletion in skeletal muscle, unlike water intake [2,3]. After prolonged exercise, exogenous carbohydrate—in particular glucose—is the primary source of glycogen synthesis, and carbohydrate intake is also important for stimulating the secretion of insulin, which is the activator of glucose uptake and glycogen synthase in skeletal muscle. Indeed, there is a positive correlation between carbohydrate intake (at <1.2 g/kg/h of carbohydrate) and the glycogen repletion rate during the post-exercise phase, whereas the consumption of an excessive amount of carbohydrates (~1.2 g/kg/h) did not seem to facilitate further skeletal muscle glycogen repletion [4]. The mechanism that underlies this saturation of post-exercise glycogen recovery in skeletal muscle is not yet known.

Similar to the plateau in post-exercise glycogen recovery, exogenous carbohydrate oxidation also hits a peak with increasing carbohydrate intake amounts [5,6]. The saturation of exogenous carbohydrate oxidation is thought to be partly due to the limited capacity of glucose transporters into enterocytes. This hypothesis is based on studies showing that the co-ingestion of different types of monosaccharides transported by different glucose transporter isoforms (e.g., glucose, transported by sodium-D-glucose co-transporter 1 (SGLT1), and fructose, transported by glucose transporter 5 (GLUT5)) resulted in a greater carbohydrate oxidation rate compared to that after the intake of a single type of carbohydrate (e.g., glucose only) [6,7]. Other functions of the gastrointestinal tract, such as gastric emptying and the digestion of polysaccharides into monosaccharides, are not considered to be limiting factors for exogenous carbohydrate oxidation [8,9]. In fact, several research groups reported that the administration of a glucose infusion after exercise stimulated skeletal muscle glycogen synthesis at much higher rates [10,11] compared to that reported in a study in which glucose was orally administered after exercise [4]. It can be postulated that post-exercise glycogen recovery after the ingestion of carbohydrate would be limited by the capacity of monosaccharide absorption in the small intestine.

An animal study indicated that the function of carbohydrate absorption in the small intestine was increased by the larger dietary carbohydrate intake within only a few days [12]. A clinical study reported that glucose supplementation 3 times/day for 10 days accelerated the gastric emptying of glucose [13]. As gastric emptying is negatively regulated in response to the length of exposure to glucose in the small intestine [14], higher carbohydrate intake would result in a stimulation of carbohydrate absorption in the gastrointestinal tract. This rapid change in the carbohydrate-absorption function after the modification of the dietary carbohydrate content seemed to be due to an altered glucose transporter level at the small intestine. It has been demonstrated that the expression of SGLT1 at the small intestine in mice was significantly increased by a high-carbohydrate (70%cal) diet for 2 weeks compared to that after an extremely low-carbohydrate (1.9%cal) diet [15]. Another study revealed that the SGLT1 protein level in piglets was significantly higher after 52.6%cal and 60.3%cal carbohydrate diets compared to after a 35.9%cal carbohydrate diet [16]. These adaptations were observed at only 3–14 days after the changes in the animals’ dietary carbohydrate composition [15,16].

SGLT1 is localized consistently at the brush border membrane (BBM, which is the area exposed to the luminal contents) and is thought to be crucial for glucose absorption into the enterocyte cells [17,18,19]. After entering the enterocyte cells, glucose is exported from across the basolateral membrane into the portal blood vessels by a facilitated diffusion via glucose transporter 2 (GLUT2) [20]. Moreover, it has been demonstrated that GLUT2 could be inserted into the BBM in response to the increased glucose concentration at luminal, and this GLUT2 translocation is thought to facilitate glucose uptake [21,22]. Collectively, the protein levels of SGLT1 and GLUT2 in enterocytes would affect glucose absorption.

Another possible influencer of post-exercise glycogen recovery is the protein level of glucose transporter 4 (GLUT4) in skeletal muscle. Human subjects with a higher GLUT4 protein level in skeletal muscle showed greater post-exercise glycogen repletion [23,24]. Higher dietary carbohydrate intake was reported to be associated with a higher skeletal muscle GLUT4 level in both humans [25,26] and rodents [27], but conflicting results have been obtained [28,29,30]. In the rodent study, the higher GLUT4 level after higher dietary carbohydrate intake was associated with a higher glycogen level in skeletal muscle [27].

We hypothesized that a habitual high-carbohydrate diet intake would stimulate post-exercise glycogen recovery by increasing the protein content of glucose transporters in the small intestine and skeletal muscle, and we conducted the present study to test this hypothesis in mice. We set the percentage of calories from carbohydrate in the diet as between 50% and 70% to compare the influences of dietary carbohydrate intake within a realistic range of habitual carbohydrate composition by humans.

## 2. Materials and Methods

### 2.1. Animals

Seven-week-old male Institute of Cancer Research (ICR) mice were purchased from CLEA Japan (Tokyo). The experimental room was kept dark at 07:00–19:00, and the room temperature was maintained at 23 °C. All experiments were performed in the dark phase when the mice were active. During acclimatization, mice were individually housed in a standard cage for 1 week. The mice consumed a standard lab chow (MF; 3.6 kcal/g; 60%cal from carbohydrate, 13%cal from fat, 27%cal from protein; Oriental Yeast, Tokyo, Japan) and tap water ad libitum. All animal experiments were conducted in accordance with the ethical standards of the Committee on Animal Care and Use, The University of Tokyo; all experimental protocols were approved by this committee (approval no. 30-6). Following the principle of a reduction of the number of animals used, we set a minimal number of mice.

### 2.2. Experimental Procedures

After the acclimatization period, we divided mice with similar average body weights into two groups: a moderate-carbohydrate diet (MCHO) group and a high-carbohydrate diet (HCHO) group. The compositions of the two diets are presented in Table 1. The HCHO diet was the D12450J diet (Research Diets, New Brunswick, NJ, USA), and the MCHO diet was a modification of the D12450J diet with a smaller amount of corn starch and a larger amount of lard.

Both groups of mice were provided their experimental diets for 10 days before the experimental day. For three consecutive days before the experimental day, mice in the exercise group were acclimatized with running on the treadmill at a speed of 25 m/min for 10 min each day. On the experimental day, after the mice were fasted for 1 h to avoid a postprandial state, the mice ran on the treadmill at 25 m/min for 60 min.

#### 2.2.1. Experiment 1

**Figure 1:**
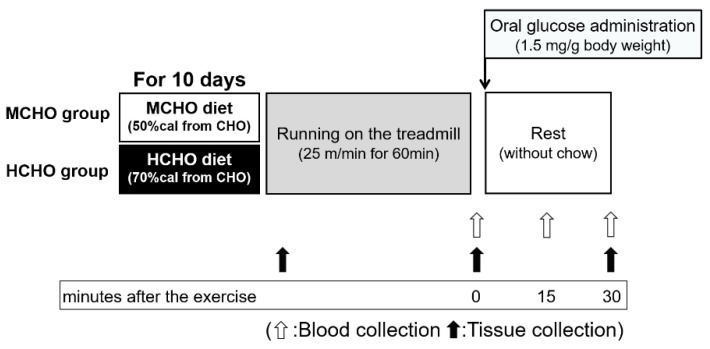
The design of Experiment 1. Eight-week-old male ICR mice were separated into two groups: those fed a high-carbohydrate diet (70%cal from carbohydrate, HCHO) group and those fed a moderate-carbohydrate diet (50%cal from carbohydrate, MCHO) group. After consuming these diets for 10 days, the mice ran on a treadmill at 25 m/min for 60 min in a fed state. Tissues were harvested at the sedentary state, immediately after the exercise, and 30 min after the post-exercise oral administration of glucose (1.5 mg/g of body weight). The experimental diet was removed at 60 min before running to avoid a postprandial state, and the diet was not provided during the post-exercise phase. Blood was collected from a tail vein immediately after the exercise (before the glucose administration) and at 15 and 30 min after the post-exercise oral glucose administration.

The design of Experiment 1 is described in Figure 1. Mice in each dietary group (moderate-carbohydrate and high-carbohydrate) were divided further into three groups with similar average body weights: a sedentary group (n = 7 or 8), an exercised group (EX, n = 7), and an exercised with post-exercise glucose administration group (EX + G, n = 7). Mice in the sedentary group were euthanized by bloodletting from the vena cava under isoflurane inhalation within 3 min, and then mucosa samples were harvested from the jejunum, blood, liver, and muscles (soleus and plantaris). Mucosa samples were scraped off from the jejunum into ice-cold buffer 1 (100 mM of mannitol, 2 mM of HEPES/Tris, pH 7.4) containing a protease inhibitor mixture (cOmplete Mini, EDTA-free; Roche Applied Science, Mannheim, Germany) and phenylmethylsulfonyl fluoride (PMSF). The samples were quickly frozen and stored at −80 °C.

Immediately after they performed the running exercise, the mice in the EX group were euthanized and the same set of samples (blood, liver, and muscles) were harvested.

The mice in the EX + G group were orally administered a glucose solution (1.5 mg/g of body weight (BW), volume of ingestion: 0.01 mL/g of BW) via a stomach tube. After the exercise was completed (before the oral glucose administration) and at 15 and 30 min after the post-exercise glucose administration, we quickly collected blood from a small cut made at the end of the mouse’s tail without anesthesia. At 30 min after the glucose administration, the EX + G mice were euthanized and the liver and muscles were harvested. The tubes containing blood samples were centrifuged at 5000×C *g* for 10 min at 4 °C, and plasma samples were collected and stored at −80 °C.

#### 2.2.2. Experiment 2

Mice in each dietary group (n = 7) were orally administered a glucose solution (1.5 mg/g of body weight (BW), volume of ingestion: 0.01 mL/g of BW) via a stomach tube. After the exercise was completed (before the oral glucose administration) and at 15 min after the post-exercise oral glucose administration, we quickly collected blood from a tail as with Experiment 1. At 15 min after the glucose administration, the mice were sacrificed under isoflurane inhalation, and the blood from the portal vein was harvested. The tubes were centrifuged at 5000× *g* for 10 min at 4 °C, and plasma samples were collected and stored at −80 °C.

### 2.3. Analytical Methods

#### 2.3.1. Blood Analysis

Blood glucose concentrations obtained from a tail vein were measured by an autoanalyzer (Glutest Ace; Arkray, Kyoto, Japan). The plasma insulin concentration was measured using an enzyme-linked immunosorbent assay (ELISA) kit (Mouse Insulin ELISA Kit; Mercodia, Uppsala, Sweden). We calculated the incremental area under the curve (iAUC) for the blood glucose concentration with the trapezoidal rule.

#### 2.3.2. Protein Isolation from Mucosa Samples and Muscles

Mucosal samples and soleus and plantaris muscles were homogenized in an ice-cold radio-immunoprecipitation assay buffer (50 mM of Tris-HCl pH 7.4, 150 mM of NaCl, 0.25% deoxycholic acid, 1% NP-40, and 1 mM of ethylenediaminetetraacetic acid (EDTA)) added with a protease inhibitor mixture and a phosphatase inhibitor mixture (PhosSTOP; Roche Applied Science). Homogenates were centrifuged at 700× *g* for 20 min at 4 °C and then the supernatants were collected. Protein concentrations of the supernatants were measured by a bicinchoninic acid assay (Thermo Fisher Scientific, Waltham, MA, USA).

#### 2.3.3. Brush Border Membrane Extraction from Jejunum Mucosa

Brush border membranes (BBMs) were isolated from mucosa of the jejunum by MgCl2 precipitation as described by Roder et al. [17] with modification. Samples were homogenized at 4 °C and then incubated with MgCl_2_ (20 mM final concentration) for 15 min. After the centrifugation at 3,000 g for 15 min at 4 °C, the supernatants were further centrifuged at 30,000× *g* for 30 min at 4 °C. The pellet was resuspended in ice-cold buffer 2 (300 mM of mannitol, 20 mM of HEPES/Tris, pH 7.4) containing a protease inhibitor mixture and PMSF. Samples were incubated with MgCl_2_ (20 mM final concentration) for 15 min. After centrifugation at 3000× *g* for 15 min at 4 °C, the supernatants were further centrifuged at 30,000× *g* for 30 min at 4 °C. The final pellets were resuspended with buffer 2.

#### 2.3.4. Western Blotting

Western blotting was performed as previously described [31]. Equal amounts of protein samples (5–10 μg for muscles and total mucosa, 2 μg for BBM) and a prestained molecular weight marker (Bio-Rad, Hercules, CA, USA) were loaded on 7.5% sodium dodecyl sulfate-polyacrylamide gel electrophoresis (SDS-PAGE) gels for 60 min at 150 V. The proteins were then transferred from the gels to polyvinylidene difluoride (PVDF) membranes using Transblot Turbo (Bio-Rad). The membranes were blocked with PVDF blocking reagent (Toyobo, Osaka, Japan) for 60 min at room temperature. After being washed, the membranes were incubated with the primary antibody in Can Get Signal Solution 1 (Toyobo) (1:1000 or 1:2000 dilution) overnight at 4 °C. After being washed, the membranes were incubated with goat-anti-rabbit IgG (American Qualex, San Clemente, CA, USA) in Can Get Signal Solution 2 (Toyobo) (1:6000 dilution) for 60 min at room temperature. The proteins were detected using Pierce ECL Western Blotting Substrate (Thermo Fisher Scientific) and visualized by a ChemiDoc system (Bio-Rad). Densitometric analyses of the captured images were performed using Bio-Rad Quantity One software ver. 4.6.1. All membranes were stained with Ponceau-S solution (P7170-1L; Sigma–Aldrich, St. Louis, MO, USA) to verify equal loading of the proteins.

The following antibodies were purchased from Cell Signaling Technology Japan (Tokyo): Akt (no. 9272), phosphorylated Akt (Thr^308^, no. 9275, Ser^473^, no. 9271), Akt substrate 160 kDa (AS160, no. 2670), phosphorylated AS160 (Thr^642^, no. 8881), glycogen synthase (GS, no. 3893), phosphorylated GS (Ser6^41^, no. 3891), hexokinase 2 (HK2, no. 2867), AMP-activated protein kinase (AMPK, no. 5832), and phosphorylated AMPK (Thr^172^, no. 2535). Antibodies against sodium-D-glucose co-transporter 1 (SGLT1, no 07-1417) and glucose transporter 2 (GLUT2, no. 07-1402-I) were purchased from Merck (Darmstadt, Germany). Antibodies against pyruvate dehydrogenase (PDH) E1α subunit (no. ab168379) and PDH E1α subunit phosphorylated at Ser^293^ (no. 177461) were purchased from Abcam (Cambridge, UK). Polyclonal antiserum specific for GLUT4 was kindly gifted from the laboratory of Dr. John O. Holloszy (Washington University, St. Louis, MO, USA).

#### 2.3.5. Glycogen Levels in Skeletal Muscles and Liver

The glycogen concentrations in the muscles and liver were measured by the phenol-sulfuric acid method [31,32]. The samples were boiled in 20 volumes of 30% KOH saturated with Na_2_SO_4_ for 10 min. After adding 99.5% ethanol (1.2 volumes of 30% KOH solution), we centrifuged the samples at 2700× *g* for 20 min at 4 °C. The pellets were dissolved in distilled water and used as sample solutions. The sample solutions were mixed with a half volume of 5% phenol, and five volumes of 98% H_2_SO_4_ were added and placed for 10 min at room temperature. The absorbance at 490 nm was read in a spectrophotometer. A standard curve was made with a glucose stock solution (cat. no. 12-0820, Sigma–Aldrich, St. Louis, MO, USA).

### 2.4. Statistical Analysis

All values are expressed as the mean ± standard error. The statistical analysis was performed by Prism 8 software (GraphPad Software, San Diego, CA, USA). We performed a two-way repeated measures analysis of variance (ANOVA) (glucose administration × diet) to analyze the blood glucose and plasma insulin concentrations of the mice after the post-exercise glucose administration. The significance of differences in the body weight, food consumption, the delta (∆) glycogen and blood glucose level, the protein levels of glucose transporters in jejunum, and the blood glucose concentration iAUC was analyzed by an unpaired *t*-test. If a normal distribution was not revealed by the Shapiro–Wilk normality test, we performed the Kruskal–Wallis test. A two-way ANOVA (glucose administration × diet) was performed to analyze post-exercise glycogen and protein contents. Statistical significance was set at *p* < 0.05.

## 3. Results

### 3.1. Body Weight and Food Consumption

The data of body weight (BW) and food consumption are summarized in Table 2. There were no significant differences in the initial or final body weights between the moderate-carbohydrate and high-carbohydrate diet groups (initial BW, *p* = 0.364; final BW, *p* = 0.318). The ∆BW (i.e., the difference between the initial and final BW for each mouse) was not significantly different between the two diet groups (*p* = 0.924), and no significant difference in the total food consumption was observed between the groups (*p* = 0.259).

### 3.2. Protein Levels of SGLT1 and GLUT2 in the Jejunum in the Sedentary State

**Figure 2:**
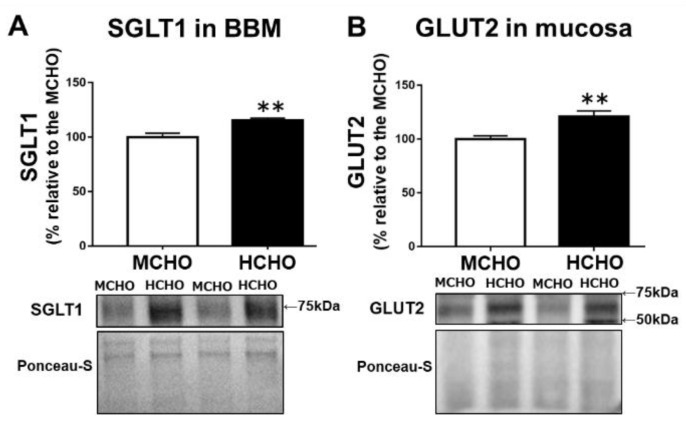
(**A**) The SGLT1 protein level in the brush border membrane (BBM) fraction extracted from jejunum mucosa and (**B**) the GLUT2 protein level in jejunum mucosa in mice fed the HCHO diet (black, n = 7) or the MCHO diet (white, n = 8) for 10 days. Values are mean ± standard error (SE). All data are shown as relative values against the MCHO group. ** *p* < 0.01 vs. the MCHO group.

Compared to the MCHO group, the HCHO group exhibited significantly higher levels of both SGLT1 protein in the BBM fraction from the jejunum mucosa (*p* = 0.003, Figure 2A) and GLUT2 protein in the jejunum mucosa (*p* = 0.004, Figure 2B) in the mice in the sedentary state.

### 3.3. Glycogen Levels in Tissues at the Sedentary State

At the sedentary state, no significant between-group difference was observed in the glycogen concentration in the soleus muscle (MCHO: 2.9 ± 0.4 mg/g wet weight [wt], HCHO: 3.3 ± 0.6 mg/g wt, *p* = 0.616) or plantaris muscle (MCHO: 3.8 ± 0.2 mg/g wet wt, HCHO: 4.2 ± 0.4 mg/g wt, *p* = 0.354). In the liver, there was no significant difference in glycogen concentration at the sedentary state between the two diet groups (MCHO: 29.3 ± 8.2 mg/g wt, HCHO: 36.4 ± 5.4 mg/g wt, *p* = 0.483).

### 3.4. Glycogen Levels in Tissues after the Post-Exercise Glucose Administration

**Figure 3:**
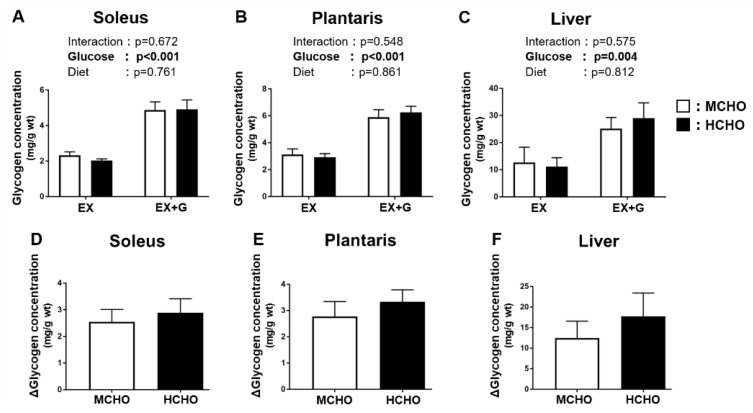
Glycogen concentrations in (**A**) soleus and (**B**) plantaris muscle and (**C**) liver of mice fed the HCHO (black) or MCHO (white) diet. Tissues were harvested immediately after treadmill running (EX) or 30 min after the post-exercise oral glucose administration (1.5 mg/g BW, EX + G). (**D**–**F**): Difference in glycogen concentration between the EX + G and EX mice in each diet group (Δglycogen concentration). Values are mean ± SE (n = 6–8).

During the post-exercise phase, a main effect of the glucose administration on the glycogen level was observed, but no significant main effect of diet was detected (Figure 3A,B). The ∆glycogen level (i.e., the difference between the EX-G and EX values in each diet group) was not significantly different between the two diet groups in the soleus or in the plantaris (soleus: *p* = 0.640, plantaris: *p* = 0.457, Figure 3D,E). A main effect of glucose administration on the liver glycogen level was revealed, but there was no significant main effect of the diet (Figure 3C). The ∆liver glycogen level (i.e., the difference between the EX-G and EX mice in each dietary group) was not significantly different between the two dietary groups (*p* = 0.464, Figure 3F).

### 3.5. Blood Glucose and Plasma Insulin Concentrations after Post-Exercise Glucose Administration

**Figure 4:**
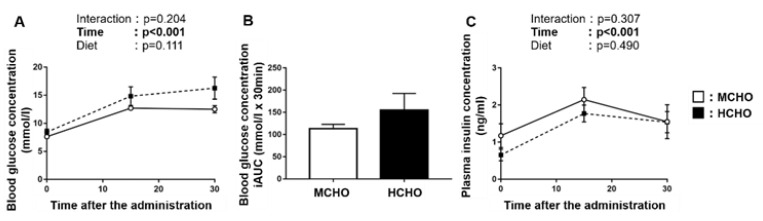
(**A**) Blood glucose, (**B**) the incremental area under the curve (iAUC) of blood glucose, and (**C**) plasma insulin concentrations after the post-exercise oral administration of the glucose solution (1.5 mg/g BW) in mice fed the HCHO (black; n = 7) or MCHO (white; n = 7) diet. Values are mean ± SE.

There was a significant main effect of time on the blood glucose concentration after the post-exercise oral glucose administration (Figure 4A), but no main effect of diet was observed. No significant difference in the incremental area under the curve of the blood glucose concentration was shown between the MCHO and HCHO groups (Figure 4B). A main effect of time on the plasma insulin concentration was demonstrated, but not an effect of the diet (Figure 4C).

### 3.6. Proteins Involved in the Carbohydrate Metabolism in Skeletal Muscle after Post-Exercise Oral Glucose Administration

**Figure 5:**
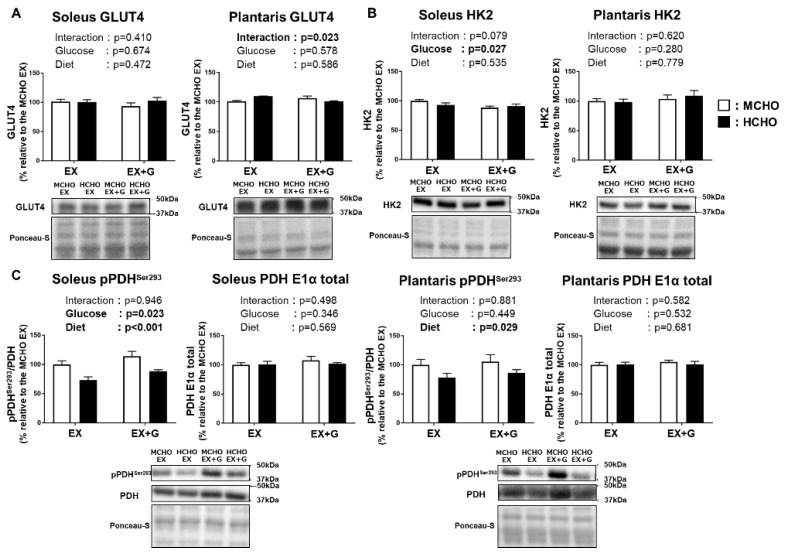
Protein levels of GLUT4 (**A**), hexokinase 2 (HK2) (**B**), and phosphorylated pyruvate dehydrogenase (PDH) E1α subunit Ser^293^ (**C**) in mice fed the HCHO (black) or MCHO (white) diet for 10 days (n = 6–8). Tissues were harvested as described in the Figure 2 legend. Values are mean ± SE. All data are shown as relative values against the MCHO EX group.

The protein level of GLUT4, a dominant isoform of glucose transporter in skeletal muscle, was not influenced by glucose or diet (Figure 5A). The protein level of hexokinase 2, a dominant isoform of the enzyme catalyzing the conversion of glucose into glucose-6-phosphate, was also not influenced by the diet (Figure 5B). A significant main effect of diet was seen on the Ser^293^ phosphorylation of pyruvate dehydrogenase (PDH), a rate-limiting enzyme of carbohydrate oxidation in mitochondria, in both soleus and plantaris muscles (Figure 5C). In the soleus, a main effect of glucose administration on PDH Ser^293^ phosphorylation was observed.

### 3.7. Phosphorylation Levels of Proteins Influenced by the Insulin Signaling Cascade in Skeletal Muscle after the Post-Exercise Oral Glucose

**Figure 6:**
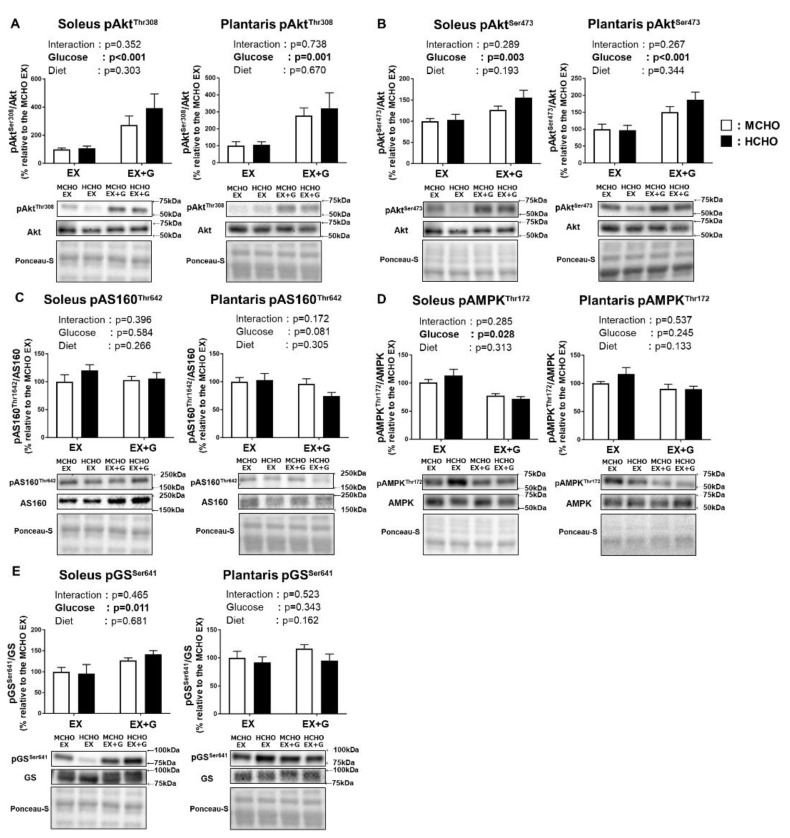
Phosphorylation levels of Akt Thr^308^ (**A**), Akt Ser^473^ (**B**), Akt substrate 160 kDa (AS160) Thr^642^ (**C**), AMP-activated protein kinase (AMPK) Thr^172^ (**D**), and glycogen synthase (GS) Ser^641^ (**E**) in mice fed the HCHO (black) or MCHO (white) diet for 10 days (n = 6–8). Tissues were harvested as described in the Figure 2 legend. Values are mean ± SE. All data are shown as relative values against the MCHO EX group.

We observed a main effect of glucose administration on the Thr^308^ and Ser^473^ phosphorylation of Akt, a proximal signaling molecule of insulin signaling, in both soleus and plantaris muscle; however, no significant main effect of diet was revealed (Figure 6A,B). The phosphorylated state of AMP-activated protein kinase (AMPK), which regulates the glucose transport across the sarcolemma in skeletal muscle in response to diverse forms of cellular stress including exercise, was not affected by diet (Figure 6C). In the phosphorylated state of AS160 Thr^642^, a downstream substrate of Akt and AMPK, a main effect of glucose administration was observed in the soleus muscle, but no significant main effect of diet was detected (Figure 6D).

In the soleus muscle of the mice, there was a tendency for the glucose administration to influence the phosphorylation of GS Ser^641^, which is one of the key regulatory sites of GS (*p* = 0.070). No significant main effect of glucose administration or diet on this parameter was shown (Figure 6E).

### 3.8. Plasma Glucose Concentrations in the Portal Vein and Tail Vein at 15 min after Post-Exercise oral Glucose Administration

**Figure 7:**
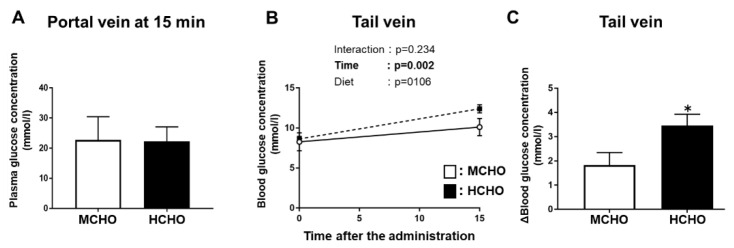
Glucose concentrations in blood obtained from the (**A**) portal vein or (**B**) tail vein after the post-exercise oral administration of the glucose solution in the mice fed the HCHO (black) or MCHO (white) diet for 10 days (n = 7–9). (**C**) Differences in tail-vein blood glucose concentrations between immediately after exercise and 15 min after glucose administration. Values are mean ± SE. * *p* < 0.01 vs. MCHO group.

Our analyses revealed that in Experiment 2, the plasma glucose concentration in the portal vein (which is the entry site of nutrients from the gastrointestinal tract) was not significantly different between the moderate- and high-carbohydrate diet groups (Figure 7A). There was a significant main effect of glucose administration on the glucose level in blood obtained from the tail vein (peripheral), but a significant main effect of diet was not present (Figure 7B). The ∆tail vein blood glucose concentration (i.e., the difference between the value at 0 min and the value at 15 min after post-exercise glucose administration) was significantly higher in the HCHO group compared to the MCHO group (*p* = 0.036, Figure 7C).

## 4. Discussion

We examined the possibility that a habitual high-carbohydrate intake would enhance glycogen recovery after endurance exercise, since it has been reported that a higher dietary carbohydrate intake increased the protein levels of SGLT1 (which is involved in glucose absorption) in small intestine [15,16] and GLUT4, a major isoform of glucose transporter in skeletal muscle [19,20,21]. Protein levels of SGLT1 and GLUT2 in the jejunum were significantly higher in mice fed the 70%cal carbohydrate diet compared to those in mice fed the 50%cal carbohydrate diet. However, the present experiments did not reveal any significant difference in the muscle or liver glycogen concentrations between mice fed the 70%cal carbohydrate diet and those fed the 50%cal carbohydrate diet.

An earlier study indicated that a diet with higher carbohydrate content increased the capacity of glucose absorption in the small intestine within only several days of the changes in dietary carbohydrate composition [12,15,16]. In the present study, increased SGLT1 and GLUT2 protein levels were present in the mouse jejunum after 10 days of 70%cal carbohydrate diet intake compared to that after 10 days of the 50%cal carbohydrate diet.

In most of the previous studies, the influence of the carbohydrate percentage in a diet on the level of glucose transporter expression were examined between immoderate conditions. For example, experiments using rodents showed that administering a 50–70%cal carbohydrate diet resulted in significantly higher SGLT1 expression in the jejunum compared to the rodents fed a typical low (5–10%cal)-carbohydrate diet [15,33,34]. Moran et al. reported that using a 52.6%cal carbohydrate diet resulted in a greater SGLT1 protein content and greater glucose uptake capacity in the jejunum compared to the use of a 35.9%cal carbohydrate diet, and that a further increase in the dietary carbohydrate content to 60.3%cal did not increase the SGLT1 level compared to that in a 52.6%cal carbohydrate diet group [16]. Our present findings revealed that a 20%cal increase in the carbohydrate composition in a diet with a moderate amount of carbohydrate (50%cal) was effective for stimulating the level of glucose transporter proteins in the small intestine of mice.

As the amount of glycogen repletion after exercise in humans seemed to be saturated when individuals consumed a huge amount of carbohydrate during the post-exercise phase [4], the possibility exists that the capacity of glucose absorption in the small intestine would affect glycogen repletion since it is one of the possible determinants of the availability of glucose, a substrate for glycogen synthesis in skeletal muscle. SGLT1 is mainly at BBM and is thought to be crucial for glucose absorption into the enterocyte cells [17,18,19]. GLUT2 mediates glucose export from across the basolateral membrane into the portal blood vessels [20]. Moreover, GLUT2 translocation to BBM in response to the increased glucose concentration at luminal is thought to facilitate glucose uptake [21,22]. Therefore, the protein levels of SGLT1 and GLUT2 in enterocytes would affect glucose absorption. However, we observed that the 10-day feeding of the HCHO diet, which induced significantly greater SGLT1 and GLUT2 protein levels in the mouse jejunum, did not stimulate post-exercise glycogen repletion compared to the MCHO diet. In Experiment 2, we measured the glucose levels in blood obtained from the portal vein at 15 min after the post-exercise glucose administration (when the glucose level in the tail vein blood reached its peak in Experiment 1). The portal vein-blood glucose level of the HCHO group was not significantly different from that of the MCHO group at 15 min after the post-exercise glucose administration. This result indicates that the levels of glucose transporter proteins in the small intestine did not influence the entrance of glucose from the intestinal tract into the portal vein after post-exercise oral glucose administration.

One possible explanation for the similar post-exercise glycogen repletion values between the MCHO and HCHO diet groups is based on the similar total GLUT4 contents in the skeletal muscles of the mice. The protein level of GLUT4 is generally considered one of the determinants of post-exercise glucose uptake and glycogen resynthesis [23,24]. Karasawa et al. demonstrated that rats that consumed a higher (79.1%cal) carbohydrate diet for 4 weeks showed higher GLUT4 protein levels and glycogen levels in the skeletal muscle compared to those consuming a moderate (59.2%cal) carbohydrate diet [27]. In the present study, we did not observe any difference in the GLUT4 protein level in mouse skeletal muscle between the MCHO and HCHO diet groups, and the habitual higher carbohydrate diet did not induce greater muscle glycogen level.

Another influential factor for post-exercise glycogen repletion is the stimulation of glucose uptake. Several studies reported that rodents consuming a higher carbohydrate diet showed a greater increase in the phosphorylation of insulin signaling molecules [35,36] and greater glucose uptake [29,36] compared to those consuming a lower carbohydrate diet. AS160 is suggested to be an important signaling molecule for the convergence of insulin- and exercise-stimulated glucose uptake during post-exercise phase via the phosphorylation at Thr^642^ [37,38]. AS160 Thr^642^ is phosphorylated by Akt, which is downstream of phosphatidylinositol 3-kinase, and is considered to be important for insulin-stimulated glucose transport [39,40]. AMPK, which is considered an energy sensor of cells, seems to indirectly stimulate AS160 Thr^642^ phosphorylation by directly phosphorylating at Ser^704^ [41,42]. We therefore examined the phosphorylated states of proteins involved in glucose uptake stimulation in skeletal muscle. However, we did not observe any significant difference in the phosphorylated states of these proteins after the oral glucose administration. These results seem to account for the similar glycogen repletion between the MCHO and HCHO diet groups.

Our present results demonstrated that 10 days of changes in the carbohydrate composition of the diet altered the phosphorylated states of PDH E1α subunit Ser^293^ in skeletal muscles of mice, and the influence of the dietary carbohydrate composition lasted for the post-exercise phase. The PDH complex catalyzes the rate-limiting step of carbohydrate oxidation (irreversible decarboxylation of pyruvate into acetyl CoA). The activity of the active form of PDH (PDHa) is regulated mainly by the phosphorylation (inactivation)/dephosphorylation (activation) of three serine residues including Ser^293^ in the PDH E1α subunit. The consumption of a lower-carbohydrate diet attenuates the activity of PDHa in skeletal muscle both at rest with insulin stimulation [43] and during exercise [44]. These influences of the change in the dietary carbohydrate composition on the activation of PDH is generally observed in only several days (~3 to 7 days) associated with an altered PDH kinase activities [45,46]. PDHa activation is suggested to stimulate glucose uptake and glycogen synthesis in response to insulin stimulation at resting state [47]. On the other hand, a reduction in PDHa activity during post-exercise phase was concomitant with glycogen recovery [48]. To date, a direct relationship between PDHa activation and glycogen recovery during a post-exercise phase has not been clarified. Our present experiments demonstrated that the higher dephosphorylated (activated) PDH-E1α Ser^293^ in the HCHO group did not lead to enhanced glycogen recovery in skeletal muscle compared to that in the MCHO group.

In Experiment 2, the increase in the tail-vein glucose concentration from the pre-administration level was significantly higher in the HCHO group compared to that in the MCHO group despite the absence of a significant difference in the glucose level in blood obtained from the portal vein. Orally ingested glucose is drained from the small intestine and then enters the portal vein. After entering the portal vein, some of the glucose passes through the liver and be released to the systemic circulation, and some of the glucose is extracted by the liver for glycogen synthesis [49]. As the glycogen level in the liver was not significantly different between our HCHO and the MCHO groups, we postulate that the greater increase in the blood glucose concentration after the post-exercise glucose administration in the HCHO diet group is due in part to hepatic glucose output. Supporting this speculation, other studies have indicated that the hepatic glucose production in the postabsorptive state was significantly higher in the subjects who consumed a high-carbohydrate diet compared with those who consumed a lower-carbohydrate diet [50,51]. However, to the best of our knowledge, it has not been established whether the habitual carbohydrate intake alters the hepatic glucose output in response to glucose administration or food intake after exercise. The effects of the pre-exercise dietary composition on post-exercise hepatic metabolism need to be addressed in detail in follow-up studies.

## 5. Conclusions

Although the high-carbohydrate diet (70% calories from carbohydrate) for 10 days increased the protein levels of glucose transporters (SGLT1 and GLUT2) involved in glucose absorption in the mouse jejunum compared to those of the moderate-carbohydrate diet (50% calories from carbohydrate) group, it was not accompanied by enhanced glycogen recovery in skeletal muscle and liver after post-exercise oral glucose administration. We observed that the blood glucose level in the portal vein, an entry site of glucose after the small intestine absorption, was not significantly different between the two pre-exercise dietary groups. The dietary carbohydrate content did not influence the levels of proteins related to glycogen synthesis and insulin signaling in response to post-exercise glucose administration in skeletal muscle.

## Figures and Tables

**Table 1:** Composition of the experimental diets.

	MCHO		HCHO	
**Energy Content**	**gram%**	**kcal%**	**gram%**	**kcal%**
Carbohydrate	54.0	50.0	67.0	70.0
Protein	22.0	20.0	19.0	20.0
Fat	14.0	30.0	4.0	10.0
**Energy density (kcal/g)**	**4.3**		**3.8**	
**Ingredient**	**gram**	**kcal**	**gram**	**kcal**
Casein	200.0	800.0	200.0	800.0
L-Cystine	3.0	12.0	3.0	12.0
Corn starch	303.3	1213.0	506.2	2025.0
Maltodextrin 10	125.0	500.0	125.0	500.0
Sucrose	68.8	275.0	68.8	275.0
Cellulose BW200	50.0	0	50.0	0
Soybean oil	25.0	225.0	25.0	225.0
Lard	110.2	992.0	20.0	180.0
Mineral mix S10026	10.0	0	10.0	0
Dicalcium phosphate	13.0	0	13.0	0
Calcium carbonate	5.5	0	5.5	0
Potassium citrate, 1 H_2_O	16.5	0	16.5	0
Vitamin mix V10001	10.0	40.0	10.0	40.0
Choline bitartrate	2.0	0	2.0	0
**Total**	**942.3**	**4057.0**	**1055.1**	**4057.0**

**Table 2:** Body weight and food consumption of mice in the moderate-carbohydrate diet (MCHO) and the high-carbohydrate diet (HCHO) groups.

	MCHO Group	HCHO Group
Initial body weight (g)	36.1 ± 0.4	37.1 ± 0.7
Final body weight (g)	36.5 ± 1.4	37.3 ± 1.0
Δ body weight (g)	0.5 ± 1.0	0.2 ± 0.6
Total food consumption (kcal)	174.1 ± 8.1	188.6 ± 8.2

Values are means ± standard error. n = 7 or 8 per group.
